# Prooxidant/Antioxidant Balance in Hypoxia: A Cross-Over Study on Normobaric vs. Hypobaric “Live High-Train Low”

**DOI:** 10.1371/journal.pone.0137957

**Published:** 2015-09-14

**Authors:** Tadej Debevec, Vincent Pialoux, Jonas Saugy, Laurent Schmitt, Roberto Cejuela, Pauline Mury, Sabine Ehrström, Raphael Faiss, Grégoire P. Millet

**Affiliations:** 1 Department of Automation, Biocybernetics and Robotics, “Jozef Stefan” Institute, Ljubljana, Slovenia; 2 Center of Research and Innovation on Sports, University Claude Bernard Lyon 1, Villeurbanne, France; 3 Institut Universitaire de France, Paris, France; 4 ISSUL, Institute of Sport Sciences, Faculty of Biology and Medicine, University of Lausanne, Lausanne, Switzerland; 5 Department of Physiology, Faculty of Biology and Medicine, University of Lausanne, Lausanne, Switzerland; 6 National School of Mountain Sports/National Ski-Nordic Centre, Prémanon, France; 7 Departmental Section of Physical Education and Sports, University of Alicante, Alicante, Spain; INIA, SPAIN

## Abstract

“Live High-Train Low” (LHTL) training can alter oxidative status of athletes. This study compared prooxidant/antioxidant balance responses following two LHTL protocols of the same duration and at the same living altitude of 2250 m in either normobaric (NH) or hypobaric (HH) hypoxia. Twenty-four well-trained triathletes underwent the following two 18-day LHTL protocols in a cross-over and randomized manner: Living altitude (P_I_O_2_ = 111.9 ± 0.6 vs. 111.6 ± 0.6 mmHg in NH and HH, respectively); training “natural” altitude (~1000–1100 m) and training loads were precisely matched between both LHTL protocols. Plasma levels of oxidative stress [advanced oxidation protein products (AOPP) and nitrotyrosine] and antioxidant markers [ferric-reducing antioxidant power (FRAP), superoxide dismutase (SOD) and catalase], NO metabolism end-products (NOx) and uric acid (UA) were determined before (Pre) and after (Post) the LHTL. Cumulative hypoxic exposure was lower during the NH (229 ± 6 hrs.) compared to the HH (310 ± 4 hrs.; *P*<0.01) protocol. Following the LHTL, the concentration of AOPP decreased (-27%; *P*<0.01) and nitrotyrosine increased (+67%; *P*<0.05) in HH only. FRAP was decreased (-27%; *P*<0.05) after the NH while was SOD and UA were only increased following the HH (SOD: +54%; *P*<0.01 and UA: +15%; *P*<0.01). Catalase activity was increased in the NH only (+20%; *P*<0.05). These data suggest that 18-days of LHTL performed in either NH or HH differentially affect oxidative status of athletes. Higher oxidative stress levels following the HH LHTL might be explained by the higher overall hypoxic dose and different physiological responses between the NH and HH.

## Introduction

It is well established that both, hypoxia [1, 2] and exercise [3, 4] elicit prooxidant/antioxidant perturbations. Augmented catecholamine production [5], mitochondrial redox potential reduction [6] and activation of xanthine oxidase pathway [7] are among the main mechanisms of hypoxia-induced reactive oxygen species overproduction. On the other hand, exercise mainly augments oxidative stress through increased production of superoxide anion (O_2_^°-^) and oxygen-derived intermediates [8] within the mitochondria as a result of activity-induced O_2_ flux increase. Combining altitude/hypoxic exposures and normoxic exercise training is the cornerstone of the “Live High Train Low” (LHTL) modality, routinely used by endurance athletes [9] and team sport players [10] in preparation for sea-level competitions. It is therefore not surprising that changes in prooxidant/antioxidant balance following LHTL protocols have previously been reported [11, 12].

In particular, 18-days of LHTL training has been shown to augment oxidative stress and decrease antioxidant capacity in elite runners [13]. Moreover, the impaired antioxidant status seems to persist for as long as two weeks after the LHTL cessation [12]. On the other hand, 13-days of LHTL did not alter prooxidant/antioxidant balance in elite swimmers [14]. Authors speculated that the lower hypoxic dose and the lower training intensity during the latter study [14] might explain the lack of significant differences in oxidative stress. Collectively, these data suggest that during a LHTL protocol, both hypoxia and exercise training modulate oxidative status in a dose-dependent manner.

To date, all studies investigating the effects of LHTL on oxidative status were performed in normobaric hypoxia (NH) [12–14]. However, growing body of evidence suggests that exposure to NH or hypobaric hypoxia (HH) at the same partial pressure of inspired O_2_ (P_I_O_2_) induces different physiological responses [15–17]. Two early studies indicated that both brief (40-min) [15] and prolonged (10-hrs) [16] exposure to NH results in higher ventilation and lower systemic hypoxemia compared to HH. Although the potential differences in several physiological responses between NH and HH are still debated [18], recent study by Faiss et al. [17] demonstrated that prolonged (24-hrs) exposure to HH results in higher oxidative stress and lower nitric oxide (NO) bioavailability compared to NH. The authors hypothesized that the differences in oxidative stress and NO between NH and HH could be due to higher normobaric hypoxic ventilatory responses [17]. Although the differences in oxidative stress and antioxidant status might significantly modulate ventilatory adaptations to LHTL [19] as well as erythropoetic response to hypoxia [20], and consequently influence performance gains, it is currently unknown whether LHTL performed in NH or HH differentially affects prooxidant/antioxidant balance of athletes.

Accordingly, this study aimed to compare the changes in prooxidant/antioxidant status of well-trained endurance athletes following 18-day LHTL protocol performed in either NH (simulated altitude of 2250 m) or HH (natural altitude of 2250 m). The training volumes and intensities as well as P_I_O_2_ were precisely matched on a daily basis between both protocols. We hypothesized that 18-days of LHTL in HH would induce a stronger alteration of oxidative stress and antioxidant status compared to the LHTL in NH.

## Materials and Methods

### Ethics statement

This study was part of a comprehensive research program investigating physiological, psychological and performance adaptions of endurance athletes to normobaric and hypobaric LHTL protocol. Performance-related results from the first phase of this project (i.e. group-design comparison of NH and HH LHTL) have been published previously [21]. The present study focused on comparing prooxidant/antioxidant responses following 18-day LHTL protocols in either NH or HH within a cross-over designed framework. The study was approved by two regional medical ethics committees for the Swiss (Commission Cantonale Valaisanne d’Ethique Médicale, CCVEM,Sion, Switzerland Agreement 051/09) and the French (Comité de Protection des Personnes EST I, Dijon, France, Agreement 2014-A00508-39) components of this project, respectively. All experimental procedures conformed to the standards set by the Declaration of Helsinki.

### Participants

Initially, twenty-seven highly trained male triathletes were recruited to participate in the study. All participants were extensively informed about the experimental procedures and gave a written informed consent prior to the start of the study. The participants were non-smokers, near sea-level residents (< 600 m) and were not exposed to altitudes above 2000 m for at last one month prior to the study. Three individuals were excluded during the initial study phase due to insufficient training volumes and fitness level. Ultimately, twenty-four participants underwent both LHTL protocols and only their data is reported in the paper. The participants’ baseline characteristics before both NH and HH LHTL protocols are outlined in Table 1.

**Table 1:** Baseline characteristics of the participants before the NH and the HH LHTL protocol.

	NH	HH
n	24	24
Age (yr)	23 ± 4	24 ± 4
Stature (cm)	178 ± 5	178 ± 5
Body mass (kg)	70 ± 5	69 ± 6
BMI (kg·m^-2^)	22 ± 2	22 ± 1
VO_2max_ (mL·kg^-1^·min^-1^)	65.9 ± 6.2	67.4 ± 6.8
HR_max_ (beats·min^-1^)	190 ± 7	187 ± 8
MAP (W)	373 ± 40	391 ± 37

Values are presented as means ± SD. NH, normobaric live-high train-low protocol; HH, hypobaric live-high train-low protocol; N, number of participants; BMI, body mass index; VO_2max_, maximal oxygen consumption; HR_max,_ maximal heart rate; MAP, maximal aerobic power during the cycle ergometer test.

### Study design

This cross-over designed study comprised of two main experimental campaigns. Before the first campaign the participants were equally assigned to either the NH (*n* = 12) or the HH (*n* = 12) LHTL protocol. A one-year wash-out period was implemented between the campaigns to avoid any carry-over effect from the first intervention. During the second campaign the participants’ conditions were crossed in order to complete the study cross-over design (e.g. participants who underwent the HH LHTL during the first campaign underwent the NH LHTL in the second one and vice-versa).

Each campaign comprised of the following three phases: 1) 24-week baseline phase–during which the training loads of all participants were monitored, 2) 3-week lead-in phase–during which all training sessions were supervised and loads monitored and 3) 18-day LHTL phase performed in either NH or HH. During the LHTL phase all participants performed supervised training sessions at altitudes between 1100–1200 m and resided at natural or simulated altitude of ~ 2250 m. Pulse oxygen saturation (SpO_2_) responses were measured during each night throughout the LHTL phase using a fingertip portable oxymeter (Wristox 3150, Nonin medical, Plymouth, USA).

Testing sessions were performed before (Pre) and immediately after (Post) the LHTL phase in a laboratory situated at an altitude of 1150 m. Both testing sessions included blood sampling to determine the alterations in prooxidant/antioxidant status and a graded exercise test to exhaustion to assess the changes in aerobic capacity and obtain the resting ventilatory parameters.

### LHTL protocol

At the start of each campaign (Pre testing) all participants resided at the Centre National de Ski Nordique et de Moyenne Montagne (CNSNMM; 1150 m altitude) in Prémanon, France. During the NH LHTL protocol the participants remained at the CNSNMM whereas during the HH LHTL the participants were transported to the altitude training facility at Fischeralp (Switzerland), located at an altitude of 2250 m. Similar diet, based on low nitrate/nitrite intake recommendations [22] was provided to participants in both condition throughout the LHTL phase.

During the LHTL phase, the participants were asked to spend a minimum of 12 hrs·day^-1^ in 1–3 person hypoxic rooms. The reduction of the O_2_ fraction (F_I_O_2_) was achieved and maintained using an oxygen extraction system (OBS, Husøysund, Norway) that delivered the oxygen-depleted air to the designated rooms. The system was calibrated before the start of the study using precision calibration gases. The levels of O_2_ and carbon dioxide were continuously monitored using designated probes in each room (OBS, Husøysund, Norway). The simulated altitude in the rooms was kept constant throughout the protocol in order to maintain a simulated altitude of 2250 m (F_I_O_2_ = 18.05 ± 0.03%; ambient pressure = 666.6 ± 3.6 mmHg; P_I_O_2_ = 111.9 ± 0.6 mmHg; simulated altitude ~ 2250 m).

The HH participants resided in the Fischeralp facility (P_I_O_2_ = 111.6 ± 0.6 mmHg; altitude = 2250 m) throughout the HH LHTL phase. The participants descended to lower altitudes (1100–1200 m) twice daily via cable car for training purposes (collectively ~ 4–6 hrs·day^-1^). The duration of the cable car transfer was 7 minutes. Upon completion of this phase, they were immediately transported back to Prémanon for the Post testing period.

### Training quantification

Two experienced coaches supervised all training sessions during the lead-in and the LHTL phases of the study. The coaches (one supervising the NH training and the other the HH training) were in continuous contact and fine-tuned the athletes training to precisely match the training loads and intensities between the protocols. The quantification of training load throughout the LHTL phase was performed using the ‘Objective Load Equivalent” (ECOs) method [23]. This method enables quantification of the training loads and calculation of ECOs values in different locomotion modes, such as in triathlon (i.e. swimming, cycling, running). Daily and weekly individual training ECOs values were calculated and subsequently pooled within the protocols to allow between-protocol comparisons.

### Biochemical analyses

Blood samples for assessment of prooxidant/antioxidant status were obtained one day before (Pre) and 24 ± 1 hrs after the last hypoxic exposure (Post) in the same laboratory situated at an altitude of 1150 m. On both occasions, five mL samples were taken from the antecubital vein and collected in the EDTA tubes (S-Monovette, Sarstedt, Nümbrecht, Germany). The samples were always obtained at the same time in the morning, with participants overnight-fasted and well rested. After the blood was centrifuged (10-min at 3500 rpm; 4°C) the obtained plasma was frozen to -80°C in 400-μL aliquots for subsequent analysis performed less than 6 months after the protocol in a blinded manner.

The plasma advanced oxidation protein products (AOPP) were determined by spectrophotometry and were calibrated with a chloramine-T solution that absorbs at 340 nm in the presence of potassium iodide. The absorbance of the reaction was read at 340 nm. AOPP concentrations were expressed as μmol·L^-1^ of chloramine-T equivalents. The intra-assay coefficient of variation (CV) was 5.4%.

Concentrations of plasma nitrotyrosine, as end product of protein nitration by ONOO^-^, were measured by ELISA. The intra-assay CV was 6.8%.

Concentrations of plasma malondialdehyde (MDA), as thiobarbituric reactive substances, were determined by extracting the pink chromogen with n-butanol and measuring its absorbance at 532 nm by spectrophotometry using 1,1,3,3-tetraethoxypropan as standard. The intra-assay CV was 2.2%.

Ferric-reducing antioxidant power (FRAP) plasma concentrations were measured at a controlled temperature (37°C) by spectrophotometry. FRAP concentrations were calculated using an aqueous solution of known Fe^2+^ concentration (FeSO_4_, 7H_2_O_2_) as standard at a wavelength of 593 nm. The intra-assay CV was 2.9%.

The plasmatic superoxide dismutase (SOD) activity was determined by the degree of inhibition of the reaction between superoxide radicals, produced by a hypoxanthine—xanthine oxidase system, and nitroblue tetrazolium. The intra-assay CV was 5.6%.

Plasmatic glutathione peroxidase (GPX) activity was determined as the rate of oxidation of NADPH to NADP+ after addition of glutathione reductase (GR), reduced glutathione (GSH) and NADPH, using H2O2 as a substrate. The intra-assay CV was 4.6%.

Catalase activity in the plasma was determined by using hydrogen peroxide (H_2_O_2_) as a substrate, and formaldehyde as a standard. Catalase activity was determined by the formation rate of formaldehyde induced by the reaction of methanol and H_2_O_2_ using catalase as enzyme. The intra-assay CV was 3.1%.

NO metabolism was quantified as the sum of nitrite and nitrate (NOx) concentrations as previously described [17]. After nitrate reduction by nitrate reductase, the fluorimetric quantification of NOx was based upon the reaction of nitrite with 2,3-diaminonaphthalene. The intra-assay CV was 5.4%.

The concentration of plasma uric acid (UA) was determined using a commercially available kit (Bio-Quant Inc., San Diego, CA, USA). The limits of detection for this assay are 1–100 μmol·L^−1^. As a product of purine metabolism during re-oxygenation the UA concentration reflects reactive oxygen species (ROS) production via xanthine oxidase pathway activation. The intra-assay CV was 0.9%.

The plasma pH was measured using the laboratory pH-meter inoLab 720 (WTW, Weilheim, Germany) with the accuracy of ± 0.004 (according to the manufacturer).

### Statistics

Data are reported as mean ± SD. Normal distribution of the data was confirmed using the Kolmogorov-Smirnov test. Dependent-samples Student’s *t*-test was used to compare baseline characteristics of the participants and hypoxic dose of each protocol. A one-way (NH vs. HH LHTL protocol) repeated measures (Pre vs. Post) ANOVA was used to test for interactions and main effects. When ANOVA analysis revealed significant F-ratio for the main effect or interaction a Tukey’s HSD *post hoc* test was employed to define specific differences. Pearson's correlation coefficient was used to define bivariate correlations between the changes in oxidative stress and antioxidant markers. Statistical significance was accepted at *P* < 0.05. All analyses were performed using Statistica 12.0 (StatSoft, Tulsa, USA).

## Results

### LHTL and training effort

The average P_I_O_2_ was identical between the NH (111.9 ± 0.6 mmHg) and the HH (111.6 ± 0.6 mmHg) LHTL protocol.

The cumulative hypoxic exposure time was lower in NH (229 ± 6 hrs.) compared to HH (310 ± 4 hrs.; *P* < 0.01).

Participants’ anthropometrical values, maximal aerobic capacity and HR as well as MAP were comparable before both LHTL protocols (Table 1). While the Pre- hypoxic SpO_2_ values were similar between the protocols, the average SpO_2_ values were higher throughout the NH LHTL (92 ± 1%) compared to HH (91 ± 1%; *P* < 0.01).

Average training loads during the LHTL phase were similar between the two protocols (NH = 226 ± 57 ECO·day^-1^; HH = 215 ± 56 ECO·day^-1^).

### Oxidative stress markers

**Fig 1:**
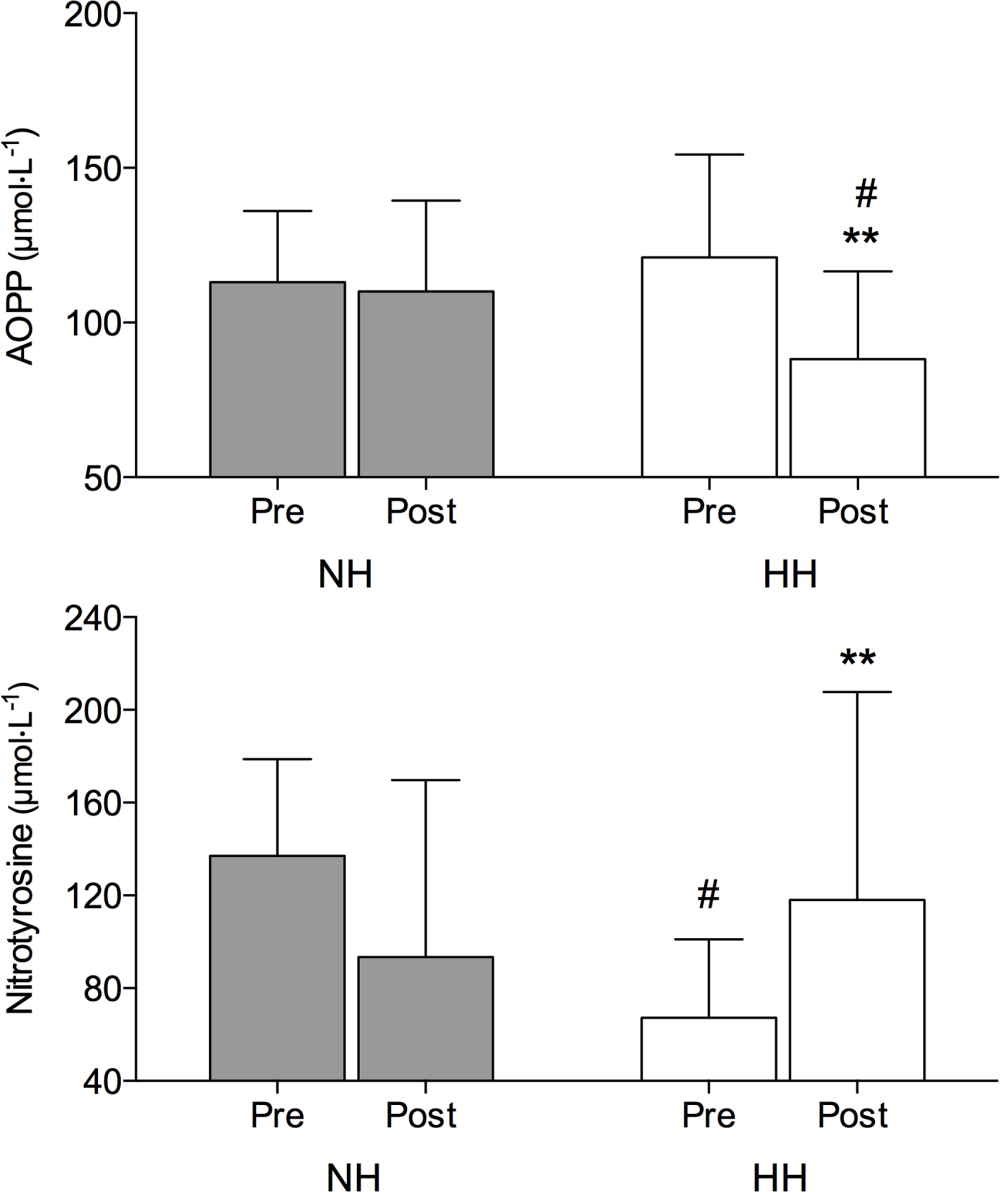
Advanced oxidation protein products (AOPP; upper panel) and nitrotyrosine (lower panel) plasma values (mean ± SD) before (Pre) and immediately following (Post) the normobaric (NH) and hypobaric (HH) LHTL protocol. Significant post hoc differences: ** *P* < 0.01 vs. Pre value; # *P* < 0.05 vs. corresponding NH LHTL value

The concentration of AOPP decreased following the HH only (-27%; *P* < 0.01) (Fig 1). At Post the AOPP was significantly higher in NH compared to HH (*P* < 0.05). As shown in Fig 1, the nitrotyrosine was significantly higher following the HH (+67%; *P* < 0.01) with no changes observed following the NH LHTL. MDA was unchanged following both, NH and HH protocol (Table 2).

**Table 2:** Plasma values of select oxidative stress and antioxidant markers obtained before (Pre) and immediately following (Post) the live-high train-low (LHTL) protocol in NH and HH.

	NH		HH	
	Pre	Post	Pre	Post
MDA (μmol·L^-1^)	5.77± 3.62	5.04 ± 4.45	5.38 ± 2.40	5.09 ± 2.01
GPX (μmol·L^-1^·min^-1^)	68.9 ± 15.2	49.1 ± 9.3**	81.2 ± 13.2#	54.3 ± 11.8**
Catalase (μmol·L^-1^)	18.9 ± 4.9	22.7 ± 5.4*	16.7 ± 6.4	15.6 ± 2.9#

Values are presented as mean ± SD. NH, normobaric live-high train-low protocol (*N* = 24); HH, hypobaric live-high train-low protocol (*N* = 24); MDA, Malondialdehyde; GPX, glutathione peroxidase. Significant post hoc differences

* *P* < 0.05

** *P* < 0.01 vs Pre value

# *P* < 0.05 vs corresponding NH value.

### Antioxidant markers

**Fig 2:**
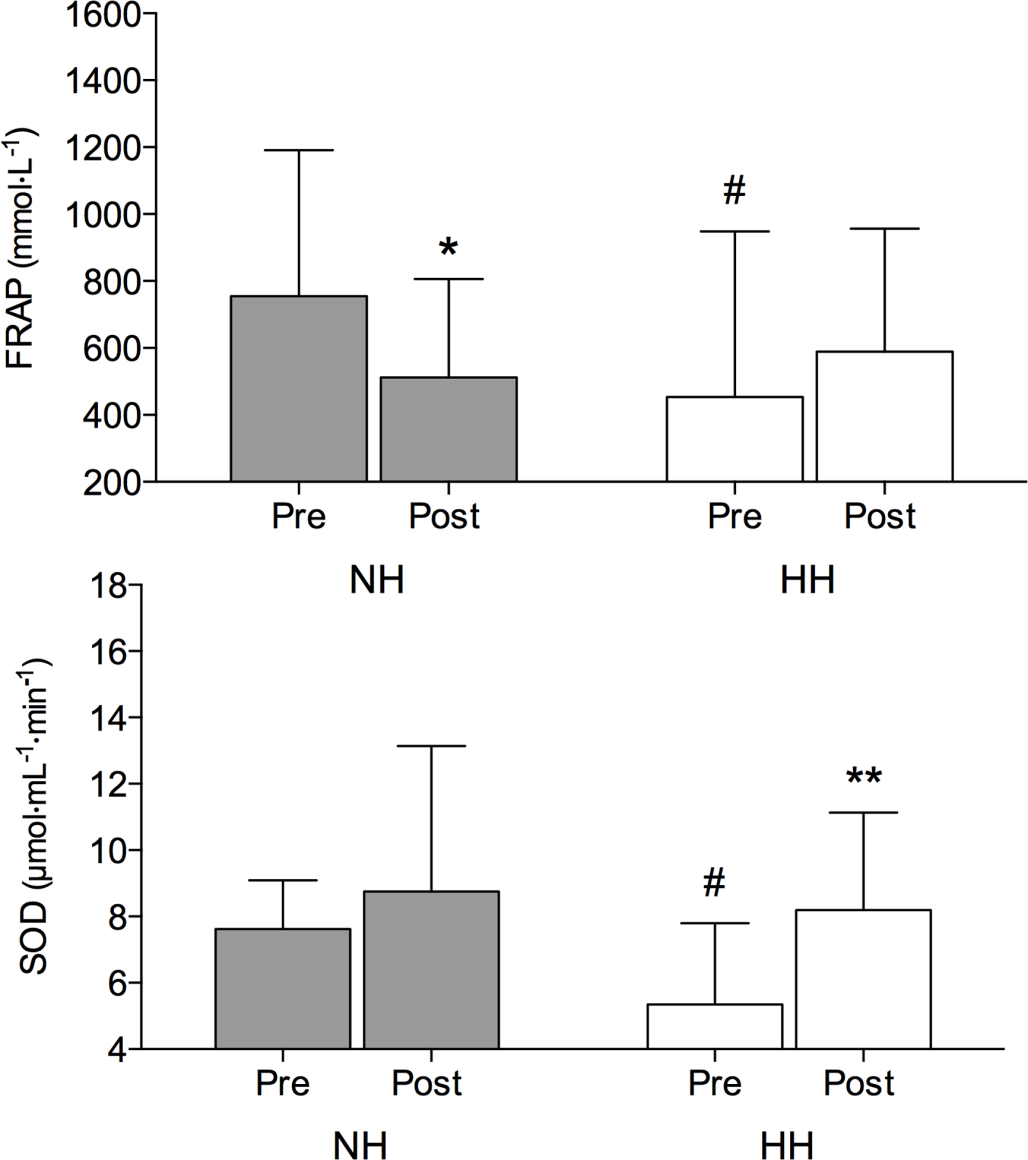
Ferric-reducing antioxidant power (FRAP; upper panel) and Superoxide dismutase (SOD; lower panel) plasma values (mean ± SD) before (Pre) and immediately following (Post) the normobaric (NH) and hypobaric (HH) LHTL protocol. Significant post hoc differences: * *P* < 0.05, ** *P* < 0.01 vs. Pre value; # *P* < 0.05 vs. corresponding NH LHTL value

Concentration of FRAP was significantly decreased following the NH (-27%; *P* < 0.05) but did not change following the HH LHTL (Fig 2). Conversely, SOD was significantly higher at Post compared to Pre following the HH (+54%; *P* < 0.01) with no changes observed following the NH LHTL (Fig 2). Compared to baseline, the GPX was decreased in both NH (-29%; *P* < 0.01) and HH (-33%; *P* < 0.01) (Table 2). Catalase activity was increased following the NH (+20%; *P* < 0.05) with no changes following the HH LHTL (Table 2).

### NOx and uric acid

**Fig 3:**
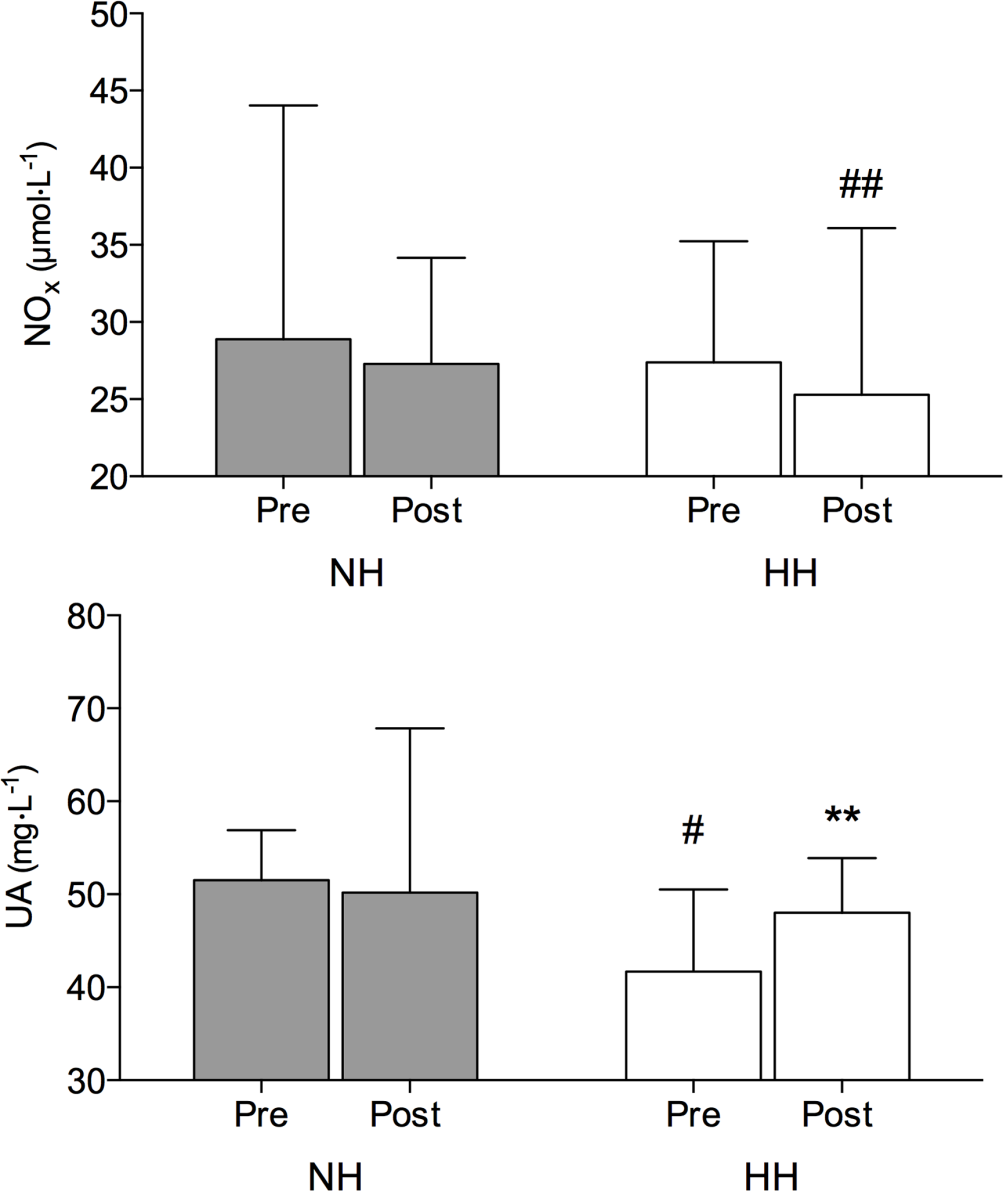
NO metabolism end-products (NOx: upper panel) and plasma uric acid (UA) values (mean ± SD) before (Pre) and immediately following (Post) the normobaric (NH) and hypobaric (HH) LHTL protocol. Significant post hoc differences: ** *P* < 0.01 vs. Pre value; # *P* < 0.05 vs. corresponding NH LHTL value; ## *P* < 0.05 vs. Pre NH LHTL value

As shown in Fig 3, the NOx levels were not significantly different following both HH and NH protocols. The UA concentration was higher after HH (+15%; *P* < 0.01) whereas no changes were detected following the NH LHTL (Fig 3).

### Plasma pH

Plasma pH decreased between Pre and Post following both NH (7.407±0.021 vs. 7.386±0.022; *P* = 0.02) and HH (7.414±0.023 vs. 7.402±0.022; *P* = 0.02) LHTL and was not significantly different between NH and HH at Post (*P* = 0.09).

### Correlations

When the data of both protocols were pooled, significant correlation was noted between the FRAP and UA (r = 0.41; *P* < 0.01) and between the percentage changes of SOD and pH (r = -0.59; *P* < 0.01)

## Discussion

We tested the hypothesis that LHTL performed in HH alters prooxidant/antioxidant balance to a greater extent than LHTL performed in NH. The main finding of the present study is that 18 days of LHTL in HH result in a higher level of oxidative stress compared to LHTL of the same duration in NH. The increased plasma levels of nitrotyrosine, SOD and UA, clearly indicate higher oxidative stress levels following LHTL in HH. On the other hand, LHTL in NH only resulted in slight reductions in GPX and increased catalase activity. This suggests that the employed combined dose of normobaric hypoxia and exercise training during the NH LHTL was insufficient to meaningfully alter prooxidant/antioxidant balance.

It has previously been shown that 18 days of normobaric LHTL increased oxidative stress and decreased antioxidant capacity in elite athletes [13], known to have superior enzymatic antioxidant defense [24]. However, the normobaric LHTL protocol in this study did not exert any significant effect on prooxidant/antioxidant balance. While reductions in GPX and FRAP as well as increased catalase activity were observed, no significant changes were noted in any of the other oxidative stress markers (i.e. MDA and AOPP) as well as nitrotyrosine and SOD. These results are in line with the findings of Pialoux et al. [14] showing that 13-days of LHTL in swimmers did not modify resting oxidative stress values.

The NH LHTL data also lend further support to the hypothesis that the benefits of repeated (moderate intensity) exercise training on enzymatic antioxidant system may counteract hypoxia-induced oxidative stress [3, 25]. Indeed, the training intensities in the present study were moderate and collectively lower than in the previous study reporting increased oxidative stress levels following 18-day NH LHTL [13]. Given that the rate of electron leakage, underlying the superoxide formation within the mitochondria respiratory chain is stable [26], higher oxygen flux, as observed during high-intensity exercise, should augment ROS production. In addition, the training modes used in this study (i.e. combining cycling, swimming and running) might also partly explain the unaltered oxidative stress status following NH LHTL. Especially, since running has been suggested to result in higher ROS production than swimming as a consequence of higher exercise-induced muscular inflammation [14]. It is also noteworthy that the daily, as well as the overall, hypoxic dose (i.e. ≥ 12 hrs·day^-1^for ≥ 18 days) employed during the NH LHTL was within the minimal recommended values for such protocols [27] and that the simulated altitude was within the recommended altitude range (2000–2500 m) to achieve optimal acclimatization responses for sea level performance [28]. Regardless of the relatively low daily hypoxic dose, the NH LHTL resulted in improved VO_2max_ and MAP and also induced significant hematological adaptations as detailed elsewhere [21]. Collectively, these results reinforce the “optimal” altitude concept [28] suggesting that preforming LHTL at altitudes between 2000–2500 m minimizes the potential detrimental effects of altitude exposures (i.e. overreaching, oxidative stress) while still providing sufficient hypoxic stimuli to enhance performance secondary to favorable hematological and ventilatory adaptations.

Contrary to the NH protocol, the participants exhibited significantly greater alterations of oxidative status following the HH LHTL. The increases in nitrotyrosine, SOD and UA clearly indicate higher oxidative stress levels following the HH LHTL. Higher SOD activity in HH vs. NH has already been demonstrated during short acute exposures [17]. The increased SOD production in HH compared to NH is also in line with the increased UA observed in this study. Indeed, the main underlying mechanism of augmented UA production in the context of intermittent hypoxia likely involves activation of the xanthine oxidase pathway that concomitantly results in increased O_2_^°-^ generation [29].

Interestingly, FRAP was unaltered following HH LHTL and was decreased as a result of LHTL performed in NH. This may be related to the increased UA levels, since UA is known to have a high antioxidant effect and is one of the main modulators of FRAP in the plasma [30]. This hypothesis is further strengthened by the significant correlation observed between FRAP and UA. The reduced levels of plasma AOPP, only observed following the HH LHTL could thus be explained by this specific UA antioxidant response.

Although lower levels of both exhaled NO [31] and plasma NOx [17] in HH vs. NH have previously been demonstrated during short-term exposures, we failed to observe a significant decrease in NOx following HH LHTL (-8%, *P* = 0.1). Nevertheless, the increased levels of nitrotyrosine associated with the observed trend for reduced NOx following the HH LHTL collectively suggest an increased ROS production during the HH protocol. This is also supported by augmented SOD activity and UA concentration. In particular, O_2_^°-^ can lead to peroxynitrite (ONOO^-^) formation via NO and ONOO^-^ can subsequently bound with tyrosine to form nitrotyrosine.

One potential explanation for the observed differences between NH and HH LHTL in oxidative stress could be the significantly lower average nocturnal SpO_2_ levels throughout the protocol in NH compared to HH LHTL protocol. Indeed, we previously reported that the arterial desaturation induced by short exposure to hypoxia exposure was positively correlated with the acute change in plasma oxidative stress [1, 2]. This finding is also in line with previous reports demonstrating lower hypoxemia levels in NH vs. HH during short-term exposures [15, 16]. While hypobaria-induced increase in dead-space ventilation has been suggested to underlie the greater hypoxemia in HH [15], other factors such as reductions in alveolar pressure and trans-capillary flux might also play a role [32]. Finally, although plasma pH was not significantly different between NH and HH at Post (trend for lower pH in HH vs. NH; *P* = 0.09), a negative correlation was noted between changes in pH and SOD. This suggests that the increased oxidative stress observed during HH as compared to NH may have been be influenced by the pH changes. Indeed, acidosis can induce oxidative stress by causing protonation of ONOO^-^ leading to radical hydroxyl generation [33, 34]. In this context, and in line with our previous results [17], we can hypothesize that the previously reported differences in ventilatory pattern between NH and HH LHTL [21] (e.g. higher nocturnal breathing frequency in HH compared to NH; suggesting lower tidal volumes during HH) could therefore explain the (relative) blood alkalosis in NH. This alkalosis observed in the NH may have reduced the ROS production and could, at least in part, explain the higher plasma oxidative stress measured in HH.

The cross-over design and a large sample size are clearly the fundamental strengths of the present study. Other important, methodological aspects of this study include: i) the study was performed on highly trained athletes, ii) training supervision during lead-in study phases ensured similar training status before the start of the respective LHTL protocols and iii) both, training and altitude were precisely matched between the NH and HH LHTL protocols.

There are nevertheless few limitations that need to be addressed. First, the cumulative hypoxic exposure time was significantly lower in NH compared to HH. Since oxidative stress alterations have been shown to follow a dose-dependent manner [20] this difference in hypoxic dose may, at least in part, explain the observed differences. It is notoriously difficult to provide the same daily hypoxic exposure during a LHTL protocol between NH and HH. This is especially true for LHTL protocols performed with elite and well-trained athletes as prolonged daily room confinement might even exert detrimental effects on physiological and psychological responses to LHTL [35]. The daily exposure times during LHTL in both NH (~ 12-hrs.) and HH (~ 16-hrs.) were in line with the majority of the other hitherto studies as detailed in a recent review [36]. Although our study may not allow us to determine the effect of barometric pressure *per se*, it reveals significant difference between two real-condition LHTL training protocols (HH and NH) in well-trained athletes in regards to oxidative stress and antioxidant responses.

Second, although the diet was not precisely measured, the same menus were used during both protocols throughout the LHTL phase. Moreover, the diet was low in nitrate/nitrite intake as recommended by Wang et al. [22] to limit the potential effects of high dietary nitrate/nitrite intakes on prooxidant/antioxidant balance. The lack of dietary intake assessment does not allow us to determine the potential effects of the non-enzymatic (dietary) antioxidants intakes. It is of note however, that at least during chronic hypoxic exposure, exercise-induced enzymatic adaptations might be more efficient for reducing the systemic oxidative stress [25] than dietary antioxidant supplementation, which has previously been shown ineffective to alleviate HH induced oxidative stress [37]. It therefore seems unlikely that the diet could explain the observed differences in oxidative stress and antioxidant status.

It is also of note that the plasma levels of selected prooxidant/antioxidant balance markers were significantly different before both protocols. In particular, the levels of Nitrotyrosine, FRAP, SOD and UA were lower, whereas the levels of GPX were higher before the HH as compared to NH LHLT. Although the participants were recruited at the same time of the yearly competitive cycle and did not seem to differ in their general health or performance status, this observation must be taken into account when interpreting these results.

Finally, the prooxidant/antioxidant balance assessment in this study was performed by measuring selected markers in the plasma. While these measures represent a valid frame for assessment of systemic oxidative stress, they do not necessarily reflect the oxidative status in other tissues and organs [38].

## Conclusions

This is the first study to date demonstrating different oxidative stress responses following two 18-days LHTL protocols; one performed in normobaric hypoxia and one in hypobaric hypoxia. The obtained data suggest that hypobaric LHTL might result in significantly higher levels of oxidative stress compared to the normobaric LHTL of the same duration. Higher oxidative stress levels following HH LHTL might be explained by the higher overall hypoxic dose and different physiological responses. Our data also suggest that hypoxia modulates oxidative stress in a dose dependent manner.

These findings may have important implications for athletes, coaches and scientists employing different LHTL protocols since parameters of performance improvement following such training modalities are closely related to oxidative stress modulation. In particular, alternations in prooxidant/antioxidant balance can significantly alter ventilatory adaptations to LHTL [19] as well as erythropoietic response to hypoxic exposure [20] and might consequently influence the purported performance benefits. Furthermore, this study also reinforces the fact that potential differences between “simulated” or “real” altitudes, both in regards to the overall hypoxic dose as well as physiological responses, need to be taken into account when utilizing these protocols for clinical or performance-enhancing purposes.
